# Correspondence: Reply to ‘Numerical modelling of the PERM anomaly and the Emeishan large igneous province’

**DOI:** 10.1038/s41467-017-00130-5

**Published:** 2017-10-10

**Authors:** N. Flament, S. Williams, R. D. Müller, M. Gurnis, D. J. Bower

**Affiliations:** 1 School of Earth and Environmental Sciences, University of Wollongong, Northfields Avenue, Wollongong, NSW 2522 Australia; 2 EarthByte Group, School of Geosciences, The University of Sydney, Sydney, NSW 2006 Australia; 3 Seismological Laboratory, California Institute of Technology, Pasadena, CA 91125 USA; 4 Department of Earth Sciences, Institute of Geophysics, ETH Zürich, Sonneggstrasse 5 8092 Zürich Switzerland; 5 Present Address: Center for Space and Habitability (CSH), University of Bern, Gesellschaftsstrasse 6, 3012 Bern, Switzerland

## Introduction

Tectonic plates and plate boundaries migrate substantially through time^1–4^ and mantle plumes are generally accepted to be mobile^5, 6^ within the convecting mantle, but it has been proposed that large low shear velocity provinces (LLSVPs) could have been fixed and rigid for as much as 540 million years (Myr)^2, 7, 8^. The hypotheses of fixed and rigid LLSVPs cannot be easily tested in the absence of constraints on the past location of lowermost mantle structures. We evaluated the hypothesis^9^ of lower mantle thermochemical structure fixity with numerical experiments. As in earlier studies^10^, we argue^11^ that the location of lower mantle thermochemical structures has changed through time.

In addition, we found that the subduction history used as boundary condition in our models^11^ produces a feature resembling the Perm Anomaly^12^. This Perm-like anomaly is the only small-scale feature consistently observed in our mantle flow models^11^, and further is consistent with the uniqueness of the Perm Anomaly across shear-wave tomography models^12^. Our models depend on initial and time-dependent boundary conditions based on tectonic reconstructions, as well as on physical parameters including the Rayleigh number and mantle rheology. We studied the sensitivity of model results to these uncertain boundary conditions and parameters^11^. We found that a separate Perm-like anomaly would form if slabs were initially inserted to >800 km depth, implying that the subduction network within which the Perm Anomaly formed between 330 and 280 Myr ago (50–100 Myr before the initial condition)^13^.

We agree with Torsvik and Domeier^14^ that “Models are representations, useful for guiding further study but not susceptible to proof”^9^. Indeed, taking models at face value rather than as representations, and attempting to disprove models without properly considering their abilities and limitations as Torsvik and Domeier^14^ do, is problematic.

**Fig. 1:**
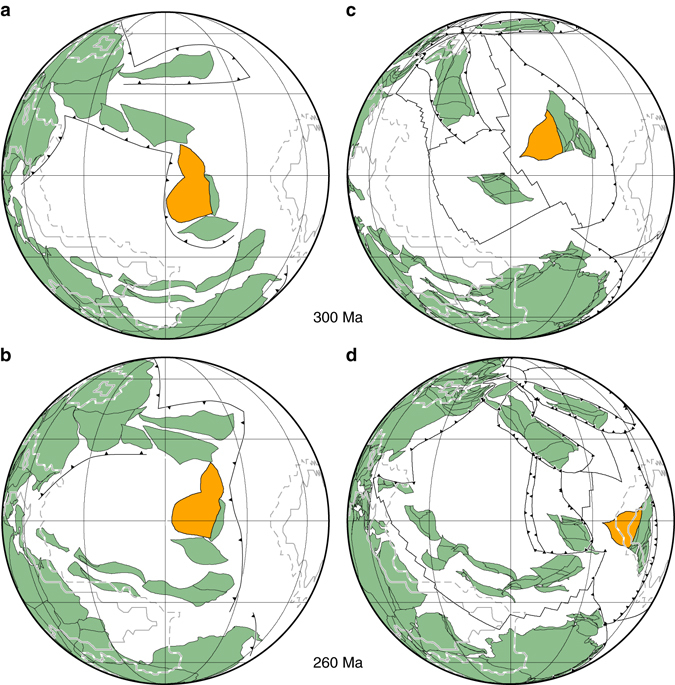
Tectonic reconstructions of the Paleotethys Ocean during the Permian Period. **a**, **b** Reconstruction of Scotese and Langford^15^. **c**, **d** Reconstruction of Domeier and Torsvik^2^. In **a** and **c**, reconstructions are shown at 300 Ma, preceding the beginning of the Permian Era by 1 Myr. In **b** and **d** reconstructions are shown at the approximate age of the Emeishan eruption (260 Ma). Continental blocks are shown in *green*, with the exception of the South China block that carries the Emeishan volcanics, shown in *orange*. The *thick black lines* in **c** and **d** are plate boundaries (mid-ocean ridges, transform faults and subduction zones), with triangles denoting the overriding plate at subduction zones. Subduction zones are drawn at their approximate location based on ref. ^15^ in **a** and **b**, as these are not available digitally. The *solid grey contour* indicates a value of five, and the dashed *grey contour* a value of one in a vote map of the location of LLSVPs in tomography models^12^. Orthographic projection centered on 90°E with graticules every 30° and the horizon at 90°

First, Torsvik and Domeier^14^ state that our models imply that the Perm Anomaly formed 190 Myr ago. We disagree. In our models that start 230 Myr ago the Perm Anomaly forms between 200 and 20 Myr ago depending on parameters^11^. Using these models as a guide, we infer that the Perm Anomaly could have formed within a long-lived, closed subduction network. Such subduction zone networks are implied in Paleozoic reconstructions^2, 15^, although their location and period of existence is uncertain and varies between reconstructions. Two closed eastern Tethyan subduction networks exist in the reconstruction model of ref. ^2^, one at high latitude around the Mongol-Okhotsk ocean between 410 and 250 Ma (a mobile Perm Anomaly could thus have existed for much of the Phanerozoic^11^), and another one reaching south to the equator between ~330 Ma (closed by 290 Ma) and 250 Ma, including the South China Block on which the Emeishan volcanics erupted close to the equator ~260 Myr ago (Fig. 1c–d). To satisfy the interpretation that the South China block should be at the western edge of the Pacific LLSVP (with the exact same location and shape than at present) during the Emeishan eruption, Domeier and Torsvik^2^ implemented a particularly complex scenario at the border between the Paleotethys Ocean and Panthalassa between 330 and 250 Myr ago (Fig. 1c–d). In contrast, earlier reconstructions^15^ located the South China block 30–40° farther west than in ref. ^2^, far away from the Pacific LLSVP, within a subduction network around the Paleotethys Ocean throughout the Permian Period (299–251 Myr ago) (Fig. 1a, b). Thus, it is clear that a connection between the Emeishan eruption and the Pacific LLSVP is not required by observation. Rather, that connection is required by the conceptual model of LLSVP stability and rigidity^2, 7, 8^, and it is therefore not an independent test of LLSVP fixity. In addition, we note that the vast majority of observations constraining the fixed LLSVP hypothesis are associated with the African LLSVP^8^ for post-Pangea times. There are no observations that suggest the Pacific LLSVP or Perm Anomaly have been fixed. However, while studies linking LIP eruptions and LLSVP edges based on observations^8^ alone have been questioned^16^, our numerical models are consistent with plumes arising from LLSVPs shaped and deformed by subduction^6^.

Second, Torsvik and Domeier^14^ base their analysis on the specific location at which the Perm-like anomaly forms in one model case, disregarding the uncertainties in model evolution related to the uncertain initial and boundary conditions^11^. The location of the subduction network in which the Perm Anomaly could have formed is uncertain and depends on tectonic reconstructions (Fig. 1). In addition, the location at which the Perm-like anomaly forms varies by several hundred kilometers depending on model parameters. We agree with Torsvik and Domeier^14^ that the high-latitude location of the Perm-like anomaly in our models is difficult to reconcile with a more equatorial location of the Emeishan volcanics ~260 Myr ago implied by paleomagnetic data, as it would require lateral migration by up to 60° at the core-mantle boundary between ~260 and 190 Myr, at an unlikely average motion rate up to ~5 cm yr^−1^ (plume ascent duration is ignored in this simple estimate). Different tectonic scenarios^15^ (Fig. 1a, b) might result in the formation of a Perm-like anomaly at lower latitude, possibly followed by northward lateral migration. We clearly identified the proposed link between the Perm Anomaly and the Emeishan volcanics as a hypothesis that remains to be tested with tectonic reconstructions that do not assume that the Emeishan LIP originated from the Pacific LLSVP^11^. This hypothesis is not central to our original article^11^ —rather, our key results are that the Perm Anomaly formed in isolation, within a long-lived, closed subduction network ~20, 000 km in length, and that it has been mobile over the last 150 Myr. These results are independent of our proposed link between the Perm Anomaly and the Emeishan volcanics, and unchallenged by Torsvik and Domeier^14^.

**Fig. 2:**
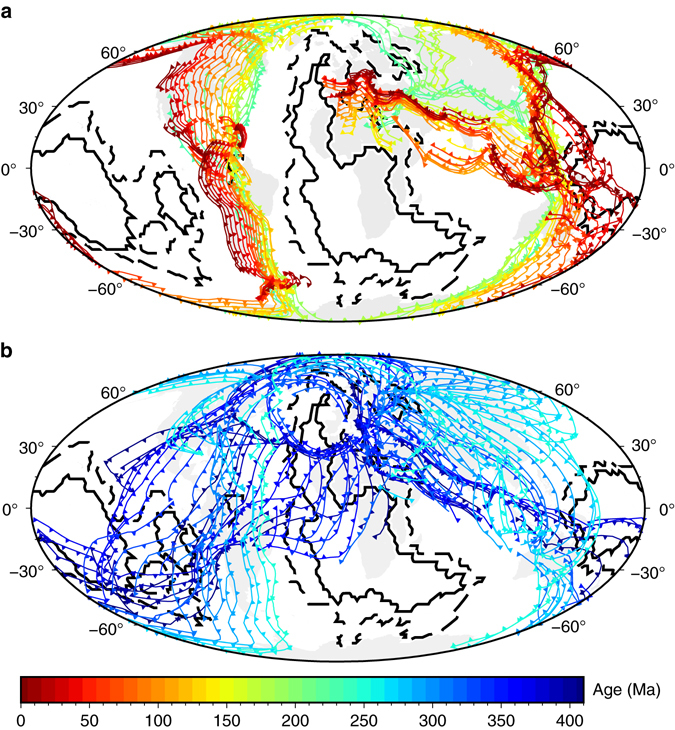
Evolution of the location of subduction zones at 10 Myr intervals in global tectonic reconstructions. **a** Between 410 and 250 Myr ago based on the reconstruction of Domeier and Torsvik^2^, in a frame of reference corrected for true polar wander. **b** Between 230 Myr ago and present based on the reconstruction of Müller et al.^4^ (reconstruction D in our original article^11^). Present-day continents are shown as *grey polygons*. The *solid black contour* indicates a value of five, and the dashed *black contour* a value of one in a vote map of the location of LLSVPs in tomography models^12^

We chose to base our analysis of deep mantle mobility on reconstructions for the last 230 Myr, a time period where plate motion histories benefit from a diverse range of independent constraints including seafloor spreading histories, hotspot trails, and seismic tomography, and resulting in relatively stable locations of subduction zones wrapping around LLSVP geometry (Fig. 2a). By contrast, reconstructions of plate boundary evolution that extend into the Paleozoic Era^2^ are based on far fewer constraints, and perhaps not coincidentally show much more rapid and less coherent changes in the location of subduction zones between 410 and 250 Myr ago, resulting in no apparent relationship between the location of subduction zones and present-day LLSVP geometry (Fig. 2b). In flow models constrained by tectonic reconstructions, lower mantle structures are shaped by subducting slabs^10, 11^. Our models are conservative in only using the last 230 Myr of plate motion history. Others^10^ have used the reconstruction of ref. ^2^ to drive such flow models and found large motion and deformation of lowermost mantle thermochemical structures during the Paleozoic Era, in contrast to relative stability of the degree 2 structure of the lower mantle over the last ~ 150 Myr. To the best of our knowledge, the alternative hypothesis that deep mantle structures could control the location of subduction zones through time^7^ remains to be tested dynamically. In the absence of such models, we note that the mobility of subduction zones implied by ref. ^2^ and their position cutting through present-day LLSVP geometry are inconsistent with the authors’ own hypotheses that stable and rigid LLSVPs^2, 8^ control the location of subduction zones through time^7^.

### Data availability

The data used in Figs 1 and 2 are available from https://www.earthbyte.org/paleomap-paleoatlas-for-gplates/, http://www.earthdynamics.org/data/Domeier2014_data.zip and ftp://ftp.earthbyte.org/Data_Collections/Muller_etal_2016_AREPS/Muller_etal_AREPS_Supplement.zip.
